# Carvedilol vs. Metoprolol Effectiveness in Patients With Left Ventricular Assist Devices: A TriNetX Analysis

**DOI:** 10.7759/cureus.87649

**Published:** 2025-07-10

**Authors:** Karldon I Nwaezeapu, Godbless Ajenaghughrure, Ekow Essien, Smith Frimpong, Chukwuemeka C Aghasili, God-dowell O Odukudu, Henna Qadri, Yash B Patel, Andrew Ndakotsu, Taraneh Zamani

**Affiliations:** 1 Internal Medicine, Trinity Health Ann Arbor Hospital, Ypsilanti, USA; 2 Internal Medicine, Good Samaritan Hospital, Cincinnati, USA; 3 Internal Medicine, Aurora Health Care, Milwaukee, USA; 4 Internal Medicine, Geisinger Health System, Wilkes-Barre, USA; 5 Internal Medicine, Delta State University, Abraka, NGA; 6 Internal Medicine, Memorial Healthcare System, Pembroke Pines, USA; 7 Internal Medicine, Medstar Health, Baltimore, USA

**Keywords:** advanced heart failure, beta blockers, carvedilol, heart failure, left ventricular assist device (lvad), lvad, mechanical circulatory support, metoprolol, mortality

## Abstract

Background: Beta-blockers are a cornerstone of heart failure management, but comparative effectiveness data for different beta-blockers in patients with mechanical circulatory support remain limited. This study aimed to compare clinical outcomes between carvedilol and metoprolol in patients with left ventricular assist devices (LVADs).

Methods: We performed a retrospective cohort study using the TriNetX Research Network (Cambridge, MA: TriNetX, LLC), a global federated health research platform providing access to electronic medical records across 104 healthcare organizations. Patients with left ventricular assist devices (ICD-10 code Z95.81) who were prescribed either carvedilol or metoprolol were identified. After propensity score matching for baseline characteristics, including cardiac and non-cardiac comorbidities, cohorts of 5,166 patients each receiving carvedilol or metoprolol were analyzed. The primary outcome was all-cause mortality. Secondary outcomes included heart failure exacerbation, cardiac arrest, cardiogenic shock, sepsis, acute kidney injury, atrial fibrillation, ventricular tachycardia, and sick sinus syndrome. Outcomes were analyzed using Kaplan-Meier survival analysis with hazard ratios (HR) and 95% confidence intervals (CI) over a one-year follow-up period.

Results: In this propensity-matched cohort study, patients receiving carvedilol demonstrated significantly lower all-cause mortality compared to the metoprolol group (15.4% vs. 17.0%; HR: 0.879, 95% CI: 0.799-0.968; p=0.009). Carvedilol was also associated with reduced incidence of cardiac arrest (5.0% vs. 6.1%; HR: 0.799, 95% CI: 0.677-0.942; p=0.007), cardiogenic shock (17.7% vs. 21.0%; HR: 0.817, 95% CI: 0.748-0.892; p<0.001), sepsis (8.8% vs. 10.4%; HR: 0.821, 95% CI: 0.724-0.930; p=0.002), and atrial fibrillation (27.3% vs. 30.7%; HR: 0.850, 95% CI: 0.792-0.914; p<0.001). However, patients in the carvedilol group experienced higher rates of heart failure exacerbation (71.3% vs. 65.9%; HR: 1.149, 95% CI: 1.097-1.204; p<0.001) and acute kidney injury (31.8% vs. 28.1%; HR: 1.132, 95% CI: 1.055-1.214; p=0.001). No significant difference was observed in the incidence of sick sinus syndrome between the two groups (8.6% vs. 8.8%; HR: 0.951, 95% CI: 0.834-1.084; p=0.450). The difference in ventricular tachycardia rates was not clinically significant despite statistical significance (23.2% vs. 22.9%; HR: 0.991, 95% CI: 0.915-1.074; p<0.001).

Conclusion: In patients with left ventricular assist devices, carvedilol was associated with lower all-cause mortality and reduced incidence of several important cardiovascular complications compared to metoprolol, despite higher rates of heart failure exacerbation and renal complications. These findings suggest that carvedilol may be preferred over metoprolol in selected LVAD patients, though individualized consideration of heart failure status and renal function remains important. Further prospective studies are warranted to confirm these findings and optimize beta-blocker selection in this high-risk population.

## Introduction

Advanced heart failure represents a significant global health burden, with increasing prevalence and high morbidity and mortality rates despite optimal medical therapy [1]. Left ventricular assist devices (LVADs) have become an established therapeutic option for patients with end-stage heart failure, serving as a bridge to transplantation, bridge to recovery, or destination therapy [2]. The expanding use of LVADs has improved survival and quality of life for many patients with advanced heart failure, though complications remain common and medical management continues to evolve [3,4].

Beta-blockers are a cornerstone of heart failure management, with established benefits in reducing mortality and morbidity in patients with reduced ejection fraction [5]. However, the optimal choice of beta-blocker in patients with LVADs remains unclear. While guidelines recommend specific beta-blockers (carvedilol, metoprolol succinate, or bisoprolol) for heart failure patients with reduced ejection fraction, these recommendations are extrapolated from studies in patients without mechanical circulatory support [6,7].

Carvedilol and metoprolol represent two commonly prescribed beta-blockers with distinct pharmacological profiles. Carvedilol is a non-selective beta-blocker with additional alpha-1 blocking and antioxidant properties, while metoprolol is a selective beta-1 adrenergic receptor antagonist [8]. These pharmacological differences could potentially impact clinical outcomes in LVAD patients, who have unique physiological considerations related to continuous-flow hemodynamics and altered neurohormonal activation [9].

Previous studies comparing carvedilol and metoprolol in heart failure patients without LVADs have shown mixed results. The Carvedilol Or Metoprolol European Trial (COMET) trial demonstrated superior survival benefits with carvedilol compared to immediate-release metoprolol tartrate [10], while other studies comparing carvedilol to extended-release metoprolol succinate have shown comparable effects on left ventricular function and clinical outcomes [11]. However, data specifically examining beta-blocker selection in LVAD recipients remain scarce.

LVAD patients face unique challenges, including altered hemodynamics, a high risk of arrhythmias, and susceptibility to right ventricular dysfunction, which may influence beta-blocker response. Additionally, the risk profiles for complications such as atrial fibrillation, ventricular tachycardia, and cardiogenic shock may differ from those of heart failure patients without mechanical support [12,13].

Given the limited comparative effectiveness data for different beta-blockers in LVAD recipients, we conducted this study to compare clinical outcomes between carvedilol and metoprolol in this unique population. Understanding the optimal beta-blocker choice for LVAD patients may help improve outcomes in this high-risk group and inform future clinical guidelines.

## Materials and methods

Data source

This study utilized data from the TriNetX Research Network (Cambridge, MA: TriNetX, LLC), a global federated health research platform providing access to electronic medical records (diagnoses, procedures, medications, laboratory values, and genomic information) across 104 healthcare organizations. The platform allows for real-time queries and robust analysis of patient data while maintaining privacy and security through de-identification. For this study, we analyzed data from the Research Network of the TriNetX platform.

Study population

We identified patients with left ventricular assist devices using the International Classification of Diseases, 10th Revision (ICD-10) code Z95.81 (presence of heart assist device). From this population, we selected patients who were prescribed either carvedilol or metoprolol after LVAD implantation.

Participants were eligible if they were aged 18 years or older at the time of LVAD implantation, had documented LVAD placement, and were prescribed either carvedilol or metoprolol after implantation with at least one year of follow-up data available. Participants were excluded if they used both carvedilol and metoprolol concurrently, underwent heart transplantation before the index date, had documented contraindications to beta-blocker therapy, were pregnant during the study period, or had incomplete clinical data.

Exposure and outcomes

The primary exposure of interest was the type of beta-blocker prescribed (carvedilol vs. metoprolol) after LVAD implantation. The primary outcome was all-cause mortality within one year after the index date. Secondary outcomes included several events within one year after the index date. These comprised heart failure exacerbation (requiring hospitalization or emergency department visit), cardiac arrest, cardiogenic shock, sepsis, acute kidney injury, atrial fibrillation (new-onset or recurrent), ventricular tachycardia, and sick sinus syndrome. All outcomes were identified using appropriate ICD-10 diagnosis codes from the electronic health records.

Covariates

To account for potential confounding, we collected multiple baseline characteristics. Demographic information included age, sex, race, and ethnicity. For cardiac comorbidities, we documented ischemic cardiomyopathy, non-ischemic cardiomyopathy, prior myocardial infarction, atrial fibrillation, ventricular tachycardia, previous cardiac arrest, valvular heart disease, pulmonary hypertension, and previous cardiac surgery. Non-cardiac comorbidities were also collected, including hypertension, diabetes mellitus, chronic kidney disease, chronic obstructive pulmonary disease, obstructive sleep apnea, stroke, peripheral vascular disease, liver disease, and malignancy. Laboratory values assessed included hemoglobin, creatinine, blood urea nitrogen, sodium, potassium, and liver function tests. We also documented concomitant medications, such as angiotensin-converting enzyme inhibitors, angiotensin receptor blockers, mineralocorticoid receptor antagonists, diuretics, amiodarone, digoxin, anticoagulants, and antiplatelet agents.

Statistical analysis

We employed propensity score matching to minimize confounding by indication and create comparable cohorts. Propensity scores were calculated using logistic regression, with the type of beta-blocker as the dependent variable and the covariates listed above as independent variables. Patients in the carvedilol and metoprolol groups were matched 1:1 using nearest-neighbor matching with a caliper width of 0.2 standard deviations of the logit of the propensity score [14].

Balance between the matched cohorts was assessed using standardized mean differences, with values <0.1 considered indicative of good balance. Baseline characteristics were compared between the matched groups using standardized mean differences and appropriate statistical tests (chi-square test for categorical variables and t-test or Mann-Whitney U test for continuous variables).

Time-to-event analyses were performed using Kaplan-Meier survival curves and Cox proportional hazards models to estimate hazard ratios (HR) with 95% confidence intervals (CI) for the primary and secondary outcomes. The proportional hazards assumption was tested using Schoenfeld residuals. Analyses were conducted using R version 4.0.3 (Vienna, Austria: R Foundation for Statistical Computing). A two-sided p-value <0.05 was considered statistically significant.

Ethical considerations

This study utilized de-identified data from the TriNetX Research Network, which complies with all relevant patient privacy regulations. The study was conducted in accordance with the Declaration of Helsinki and approved by the institutional review board. As the study used only de-identified data, informed consent was waived.

## Results

Before propensity score matching, 5,300 patients with LVADs who were prescribed carvedilol and 14,324 patients prescribed metoprolol met the inclusion criteria. Following propensity score matching, 5,166 patients remained in each group. The baseline characteristics of the matched cohorts are presented in Table 1. The matched groups were well-balanced in terms of demographics, comorbidities, and concomitant medications, with all standardized mean differences <0.1.

**Table 1:** Baseline conditions of patients in the study. P-values were calculated using the chi-square test for categorical variables. Statistical significance was set at p<0.05.

Characteristic	Carvedilol group (n=5,166)	Metoprolol group (n=5,166)	p-Value
Cardiac conditions			
Heart failure	78.8%	77.5%	0.105
Atrial fibrillation	40.6%	39.7%	0.356
Dilated cardiomyopathy	21.1%	20.5%	0.452
Other cardiomyopathies	40.4%	38.3%	0.028
Non-cardiac comorbidities			
Hypertension	59.5%	56.6%	0.003
Diabetes mellitus	41.6%	40.6%	0.308
Chronic kidney disease	16.6%	15.6%	0.164

The cohorts were well-matched for key cardiac conditions, including heart failure (78.8% vs. 77.5%; p=0.105), atrial fibrillation (40.6% vs. 39.7%; p=0.356), dilated cardiomyopathy (21.1% vs. 20.5%; p=0.452), and other cardiomyopathies (40.4% vs. 38.3%; p=0.028). Important non-cardiac comorbidities were also similarly distributed between groups, including hypertension (59.5% vs. 56.6%; p=0.003), diabetes mellitus (41.6% vs. 40.6%; p=0.308), and chronic kidney disease (16.6% vs. 15.6%; p=0.164).

Primary outcome

**Figure 1:**
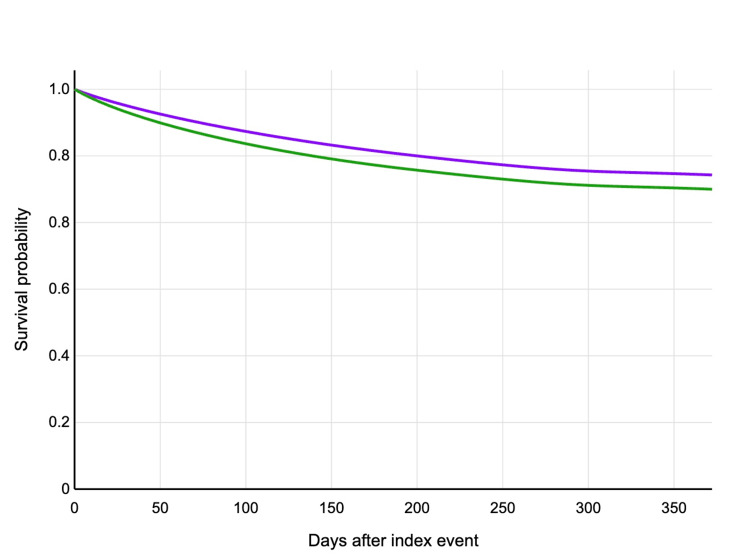
Kaplan-Meier curve showing survival probability in a year after index event. Purple line represents carvedilol and green line represents metoprolol.

The primary outcome of all-cause mortality within one year occurred in 795 patients (15.4%) in the carvedilol group and 879 patients (17.0%) in the metoprolol group (HR: 0.879, 95% CI: 0.799-0.968; p=0.009) (Figure 1). The survival benefit associated with carvedilol became apparent after approximately three months and persisted throughout the follow-up period.

Secondary outcomes

The incidence of secondary outcomes in both groups is presented in Table 2. Carvedilol was associated with a significantly lower risk of several important clinical outcomes. The incidence of cardiac arrest was lower in the carvedilol group compared to the metoprolol group (257 {5.0%} vs. 314 {6.1%}; HR: 0.799, 95% CI: 0.677-0.942; p=0.007). Similarly, cardiogenic shock occurred less frequently in patients receiving carvedilol (915 {17.7%} vs. 1,084 {21.0%}; HR: 0.817, 95% CI: 0.748-0.892; p<0.001). The carvedilol group also demonstrated reduced rates of sepsis (457 {8.8%} vs. 536 {10.4%}; HR: 0.821, 95% CI: 0.724-0.930; p=0.002) and atrial fibrillation (1,411 {27.3%} vs. 1,584 {30.7%}; HR: 0.850, 95% CI: 0.792-0.914; p<0.001).

**Table 2:** Primary and secondary outcomes of patients in this study. Statistical analysis performed using Cox proportional hazards models. Statistical significance was set at p<0.05.

Outcome	Carvedilol group (n=5,166)	Metoprolol group (n=5,166)	Hazard ratio (95% CI)	p-Value
Primary outcome				
All-cause mortality	795 (15.4%)	879 (17.0%)	0.879 (0.799-0.968)	0.009
Secondary outcomes				
Heart failure exacerbation	3,684 (71.3%)	3,405 (65.9%)	1.149 (1.097-1.204)	<0.001
Cardiac arrest	257 (5.0%)	314 (6.1%)	0.799 (0.677-0.942)	0.007
Cardiogenic shock	915 (17.7%)	1,084 (21.0%)	0.817 (0.748-0.892)	<0.001
Sepsis	457 (8.8%)	536 (10.4%)	0.821 (0.724-0.930)	0.002
Acute kidney injury	1,645 (31.8%)	1,454 (28.1%)	1.132 (1.055-1.214)	0.001
Atrial fibrillation	1,411 (27.3%)	1,584 (30.7%)	0.850 (0.792-0.914)	<0.001
Ventricular tachycardia	1,199 (23.2%)	1,181 (22.9%)	0.991 (0.915-1.074)	<0.001
Sick sinus syndrome	446 (8.6%)	454 (8.8%)	0.951 (0.834-1.084)	0.450

However, patients in the carvedilol group experienced significantly higher rates of certain adverse outcomes. Heart failure exacerbation was more common in the carvedilol group (3,684 {71.3%} vs. 3,405 {65.9%}; HR: 1.149, 95% CI: 1.097-1.204; p<0.001), as was acute kidney injury (1,645 {31.8%} vs. 1,454 {28.1%}; HR: 1.132, 95% CI: 1.055-1.214; p=0.001). No significant difference was observed in the incidence of sick sinus syndrome between the two groups (446 {8.6%} vs. 454 {8.8%}; HR: 0.951, 95% CI: 0.834-1.084; p=0.450). The difference in ventricular tachycardia rates was not clinically significant despite statistical significance (1,199 {23.2%} vs. 1,181 {22.9%}; HR: 0.991, 95% CI: 0.915-1.074; p<0.001).

## Discussion

In this large propensity-matched cohort study of LVAD recipients, we found that carvedilol was associated with significantly lower all-cause mortality compared to metoprolol. Additionally, carvedilol use was associated with reduced risks of cardiac arrest, cardiogenic shock, sepsis, and atrial fibrillation. However, these benefits came with increased risks of heart failure exacerbation and acute kidney injury. To our knowledge, this is the largest study comparing the effectiveness of different beta-blockers specifically in LVAD patients.

Our findings regarding reduced mortality with carvedilol align with some, but not all, previous studies in the general heart failure population. The COMET trial, which compared carvedilol to immediate-release metoprolol tartrate in heart failure patients without LVADs, demonstrated a 17% relative risk reduction in all-cause mortality with carvedilol [10]. However, the COMET trial has been criticized for using lower than target doses of metoprolol and employing the immediate-release formulation rather than the extended-release formulation typically used in heart failure [15]. Other studies comparing carvedilol to metoprolol succinate have shown similar effects on left ventricular function and clinical outcomes [16].

The survival benefit observed with carvedilol in our study may be explained by several mechanisms. Carvedilol's additional alpha-1 blocking effects can reduce afterload and improve peripheral circulation, which may be particularly beneficial in LVAD patients with altered hemodynamics. Furthermore, carvedilol's antioxidant properties may help mitigate oxidative stress and inflammation, which are elevated in heart failure and potentially exacerbated by the mechanical circulatory support interface [17,18].

The additional alpha-blocking and antioxidant properties of carvedilol may contribute to this benefit by reducing atrial stretch, inflammation, and oxidative stress - all of which are implicated in the pathogenesis of atrial fibrillation [19].

The reduction in cardiogenic shock associated with carvedilol is particularly relevant in LVAD patients, who remain at risk for right ventricular failure and cardiogenic shock despite left ventricular support [20]. Carvedilol's vasodilatory effects may help maintain right ventricular-pulmonary artery coupling and reduce pulmonary vascular resistance, potentially preserving right ventricular function better than selective beta-blockade alone [21].

The finding of reduced sepsis in the carvedilol group is intriguing and warrants further investigation. It is possible that carvedilol's anti-inflammatory and antioxidant properties may modulate the systemic inflammatory response, potentially reducing susceptibility to or severity of infections [22]. LVAD patients are at high risk for infections, and any intervention that reduces this risk could have significant clinical implications [23].

However, the increased risk of heart failure exacerbation and acute kidney injury observed with carvedilol requires careful consideration. The increased risk of acute kidney injury could be attributed to carvedilol's greater vasodilatory effects, which might induce hypotension and subsequently lead to a pre-renal acute kidney injury in susceptible individuals. These findings emphasize the importance of individualized beta-blocker selection based on patient characteristics and comorbidities.

Several limitations of our study should be acknowledged. First, despite propensity score matching, residual confounding may exist due to unmeasured variables. Second, the observational nature of the study precludes causal inferences. Third, we could not assess adherence to prescribed medications beyond prescription fills, which may affect the observed outcomes. Fourth, the TriNetX database may not capture all clinical outcomes if patients received care outside the participating healthcare systems. Fifth, we could not comprehensively evaluate hemodynamic parameters or device settings that might influence outcomes. Finally, while we included both metoprolol succinate and tartrate formulations, differences between these formulations might impact the comparison with carvedilol. Despite these limitations, our study has important strengths, including the large sample size specific to LVAD recipients, robust propensity matching, and comprehensive assessment of clinical outcomes.

## Conclusions

In this large propensity-matched study of LVAD recipients, carvedilol was associated with lower all-cause mortality and reduced incidence of cardiac arrest, cardiogenic shock, sepsis, and atrial fibrillation compared to metoprolol, despite higher rates of heart failure exacerbation and acute kidney injury. These findings suggest that carvedilol may be the preferred beta-blocker for many LVAD patients, though individualized consideration of heart failure status, renal function, and right ventricular function remains important for optimizing outcomes.

Future prospective randomized trials specifically in LVAD recipients are needed to confirm these findings and better define the optimal beta-blocker choice for this unique and growing patient population. Additionally, mechanistic studies exploring the physiological effects of different beta-blockers in the context of continuous-flow mechanical circulatory support would enhance our understanding of the observed clinical differences.
